# Multiple Transcripts from a 3′-UTR Reporter Vary in Sensitivity to Nonsense-Mediated mRNA Decay in *Saccharomyces cerevisiae*

**DOI:** 10.1371/journal.pone.0080981

**Published:** 2013-11-18

**Authors:** John M. Zaborske, Bethany Zeitler, Michael R. Culbertson

**Affiliations:** Laboratory of Genetics and Laboratory of Cell and Molecular Biology, University of Wisconsin, Madison, Wisconsin, United States of America; The John Curtin School of Medical Research, Australia

## Abstract

Nonsense-mediated mRNA decay (NMD) causes accelerated transcript degradation when a premature translation termination codon disrupts the open reading frame (ORF). Although endogenous transcripts that have uninterrupted ORFs are typically insensitive to NMD, some can nonetheless become prone to NMD when translation terminates at out-of-frame premature stop codons. This occurs when introns containing stop codons fail to be spliced, when translation of an upstream ORF (uORF) terminates in the 5′-untranslated region (5′-UTR) or the coding region, or when the 5′-proximal AUG initiation codon is bypassed and translation initiates at a downstream out-of-frame AUG followed by a stop codon. Some 3′-untranslated regions (3′-UTRs) are also known to trigger NMD, but the mechanism is less well understood. To further study the role of 3′-UTRs in NMD, a reporter system was designed to examine 3′-UTRs from candidate genes known to produce NMD-sensitive transcripts. Out of eight that were tested, the 3′-UTRs from *MSH4* and *SPO16* caused NMD-dependent mRNA destabilization. Both endogenous genes produce multiple transcripts that differ in length at the 3′ end. Detailed studies revealed that the longest of six reporter *MSH4-3*′*-UTR* transcripts was NMD-sensitive but five shorter transcripts were insensitive. NMD-dependent degradation of the long transcript required Xrn1, which degrades mRNA from the 5′ end. Sensitivity to NMD was not associated with extensive translational read-through past the normal stop codon. To our knowledge, this is the first example where multiple transcripts containing the same ORF are differentially sensitive to NMD in *Saccharomyces cerevisiae*. The results provide a proof of principle that long 3′-UTRs can trigger NMD, which suggests a potential link between errors in transcription termination or processing and mRNA decay.

## Introduction

Nonsense mediated mRNA decay (NMD) is an mRNA quality control mechanism that was first discovered in *Saccharomyces cerevisiae* and was later found to be ubiquitous throughout eukaryotes [1], [2], [3]. NMD prevents the accumulation of truncated proteins produced from defective transcripts. Base substitutions cause premature termination of translation whenever a sense codon is changed to a stop codon. In AT-rich genomes, multiple stop codons reside in all of the alternate reading frames of virtually every gene. For this reason, most frameshift mutations bring a premature termination codon (PTC) into register. NMD monitors the translatable RNA population through a process called RNA surveillance, resulting in the elimination of RNAs that, because of a coding error, could produce potentially deleterious truncated proteins. Splicing errors can also result in the inclusion of a PTC in the coding region, which targets the mis-spliced transcript for decay by the NMD pathway.

In *S. cerevisiae*, nonsense transcripts are targeted for NMD by a surveillance complex consisting of five proteins: Upf1, Upf2, Upf3, eRF1 and eRF3 [4], [5], [6]. Upf1 is part of a superfamily 1 ATP-dependent RNA helicase that localizes to the cytoplasm [7]. When ribosomes encounter a premature stop codon, Upf1 interacts with eRF1 and eRF3 to terminate translation [5], [8]. Upf2 and Upf3, which are involved in selective recruitment of nonsense transcripts into the NMD pathway, form a separate sub-complex [9]. Each of the two sub-complexes can interact with nonsense transcripts independently but both must be present for the surveillance complex to form and trigger NMD.

In yeast, the predominant mode of NMD-mediated transcript degradation occurs when Upf1 interacts with the decapping complex [10]. mRNA remodeling is believed to expose the cap, which triggers cap removal without the usual requirement for shortening of the poly(A) tail. Upf1 associates with ribosomes and plays a role in translation termination at PTCs as a prelude to remodeling, de-capping, and decay [11]. The mRNA is then degraded from the 5′ end by the exonuclease Xrn1. In a slower alternative pathway for NMD-mediated degradation, Upf1 interacts with Ski7, a component of cytoplasmic exosomes [12]. Following enhanced degradation of the poly(A) tail to 7–20 A residues, cytoplasmic exosomes degrade the body of the mRNA from the 3′ end [12].

In addition to RNA surveillance, NMD also affects the accumulation of transcripts produced from 458 endogenous genes in *S. cerevisiae* containing ORFs that are uninterrupted by an in-frame PTC [13]. 220 of these mRNAs are direct targets of NMD where the changes in accumulation are caused by a change in the decay rate. The remaining transcripts that show NMD-dependent changes in accumulation are affected indirectly and show no change in decay rate.

Three mechanisms have been described that trigger nonsense-mediated decay of direct targets. Two of these bring out-of-frame stop codons into register where they are recognized as PTCs that trigger NMD. Translation of upstream open reading frames (uORF) can trigger NMD when uORF termination occurs either in the 5′ leader or at an out-of-frame stop codon within the primary ORF [13], [14]. Alternatively, if translation initiation is inefficient at the normal start codon, typically the first AUG, ribosomes bypass the first AUG and continue scanning to the next AUG. If the second AUG is out-of-frame, ribosomes initiate translation in an alternative reading frame and terminate translation at an out-of-frame premature stop codon [13], [15]. In the third mechanism, some transcripts are targeted for NMD by the 3′-untranslated region (3′-UTR) [16], [17]. Although the details are poorly understood, it has been shown that transcripts with unusually long 3′-UTRs are prone to NMD, which might cause the normal stop codon to be recognized as a PTC [16], [17].

In this study we focused on the role of the 3′-UTR in NMD. We developed a reporter system to screen for 3′-UTRs that are required to trigger NMD. 3′-UTRs from two genes known to be targets of NMD were identified. In both cases, the genes produce heterogeneous transcripts that differ in the locations of the 3′ ends. Our results show that multiple transcripts containing the same ORF can be differentially susceptible to NMD. The length of the 3′-UTR appears to be the determining factor. While alternative 3′-UTR splicing in mammals has been shown to lead to variations in NMD sensitivity, to our knowledge this is the first known example of 3′-UTR variability altering NMD sensitivity in budding yeast suggesting this mechanism may be used across the domain Eukaryota [18].

## Materials and Methods

### Yeast strains

Experiments were performed using strains W303A (MATa ura3-1 his3-11,15 leu2-3,112 trp1-1 ade2-1 can1-100 upf1-?2:URA3); AAY320 (MATa leu2-3,112 trp1-1 can1-100 ura3-1 ade2-1 his3-11,15), and BY4741 (MATa his3Δ1, leu2 met15 ura3), the parent strain used to create the yeast knockout collection. Strains W303A and AAY320 was the parental control in experiments involving XRN1 and SKI7 knockouts. In some experiments, NMD-dependent changes in mRNA accumulation and decay were measured in derivatives of strain W303A because it was shown that the magnitudes of NMD-dependent changes are greatest in this strain [19].

5′ and 3′ mRNA decay pathways were analyzed using Nmd^+^ and Nmd^−^ strains carrying null alleles of *XRN1* and *SKI7* as follows: BZY18 (*MAT* ***a*** *ura3-1 his3-11,15 leu2-3,112 trp1-1 ade2-1 can1-100 upf1-Δ2:URA3 ski7:ADE2*); BZY19 (*MAT* ***a*** *ura3-1 his3-11,15 leu2-3,112 trp1-1 ade2-1 can1-100 upf1-Δ2:URA3 xrn1:ADE2*); BZY20 (*MAT*a *leu2-3,112 trp1-1 can1-100 ura3-1 ade2-1 his3-11,15 ski7:ADE2*); and BZY21 (*MAT*a *leu2-3,112 trp1-1 can1-100 ura3-1 ade2-1 his3-11,15 xrn1:ADE2*).

### Criteria for selecting candidate genes for the 3′-UTR screen

To identify genes whose transcripts are most likely targeted for NMD through the 3′-UTR, we analyzed time-course microarray data describing mRNA half-lives that were used previously to identify and classify NMD targets [13]. Out of 458 NMD-sensitive mRNAs, 238 were excluded as candidates for 3′-UTR targeting because they were indirect targets, exhibiting altered abundance but unchanged half-lives in the absence of NMD. Among the 220 direct targets, ∼75 were predicted to be targeted for NMD due to a translatable uORF and ∼50 due to bypass of the first AUG. Both invoke out-of-frame stop codons to terminate translation prematurely, and some transcripts might be targeted by both mechanisms. Among the direct targets of NMD, ∼100 candidates mRNAs remained that could be targeted by an alternative mechanism, possibly one that involves the 3′-UTR. We settled on a relatively small number of candidates because of time constraints in constructing reporters to test the 3′-UTRs for a role in NMD. Eight candidates (*MSH4, SPO16, HMO1, PET18, YMR114C, CNN1, STE2*, and *DAL5*), were selected for analysis. The 3′-UTRs from *PGA1*, which is known to be targeted for NMD through the 3′-UTR, and *ACT1*, which is known to be insensitive to NMD, were included as positive and negative controls, respectively [16].

For each candidate, an estimate was required for the length of the 3′-UTR before constructing reporters. Unlike mammals, *S. cerevisiae* genes lack a conserved transcription termination and polyadenylation signal sequence. We examined tiling microarray array data to estimate lengths of the 3′-UTRs [20]. Centromeric reporter plasmids were used to screen the effects of the 3′-UTRs on mRNA accumulation. The reporters express a hybrid gene inserted in plasmid pRS313 consisting of the *CUP1* transcriptional promoter (copper metallothionine) fused to the ORF coding for green fluorescent protein (GFP) followed by a 3′-UTR of choice.

3′-UTRs from candidate genes were inserted into the *CUP1-GFP* reporter between *Eco*RI and *Sac*I sites such that the GFP stop codon and downstream sequences were displaced by an insert containing the stop codon and 3′-UTR of the candidate gene. The plasmids containing 3′-UTRs were pJZ2 (753-nt 3′-UTR from *PGA1*); pJZ3 (345-nt from *HMO1*); pJZ4 (86-nt from *PET18*); pJZ5 (63-nt from *YMR114C*); pJZ6 (290-nt from *ACT1*); pJZ7 (133-nt from *SPO16*); pJZ9 (77-nt from *CNN1*); pJZ10 (227-nt from *MSH4*); pJZ11 (272-nt from *STE2*); and pJZ12 (1520-nt from *DAL5*).

### Translational read-through assay

To analyze translational read-through of *MSH4* transcripts, plasmids expressing the *CUP1-GFP-MSH4-3*′*-UTR* reporter were constructed that contain a FLAG epitope tag inserted following the TAA stop codon (pJZ13) and FLAG combined with a mutation that changes the TAA stop codon to a TTA leucine codon (pJZ14). pJZ15 and pJZ16 are multi-copy versions of pJZ13 and pJZ14, respectively, that carry the 2 µ origin of replication.

### RNA methods

RNA was isolated from saturated overnight cultures of cells diluted to OD_600_  =  0.1 and to grown to midlog phase, OD_600_  =  0.4–0.5. During outgrowth the *CUP1* promoter was induced with 100 µM CuSO_4_ for 1.5–3 h. To assay mRNA accumulation, 20 ml of cells were centrifuged for 3 min at 3200G after which the cells were resuspended in 0.7 ml RNA lysis buffer (10 mM Tris-Cl pH 7.4, 10 mM EDTA, 0.5% SDS) and flash frozen in a dry ice ethanol bath. RNA was prepared by adding 800 µl of acid saturated phenol pH 4.3 (Fisher) and 150 µl of RNA lysis buffer to frozen cell pellets. To assay mRNA decay, transcription was inhibited prior to RNA isolation by the addition of 25 µg/ml thiolutin (a gift from Pfizer, part number CP-4092) [13]. RNA was pelleted and flash frozen at indicated time intervals. RNA extracted at time intervals following the addition of thiolutin was prepared by adding 750 µl of acid saturated phenol and 600 µl of RNA lysis buffer to frozen cell pellets. RNA samples used to assay mRNA accumulation and decay were incubated at 65°C for 45 min with vortexing every 10–15 min. Samples were centrifuged at 4°C in a microcentrifuge at maximum speed for 15 min to pellet debris. The aqueous phase was subjected to a second acid phenol extraction. Residual phenol was removed from the aqueous phase by vortexing with 750 µl chloroform for 2 min and centrifuging for 2 min. The RNA was precipitated by adding NaOAc pH 5.3 to a final concentration of 0.3 M, 2.5 volumes of 100% ethanol and pelleted by centrifugation for 30 min at 4°C. Pellets were washed with 70% ice cold ethanol, dried, and resuspended in TE buffer.

RNA was fractionated on a 1% agarose gel and transferred to GeneScreen Plus (PerkinElmer Life Sciences). Transcripts were analyzed by northern blotting using sequence-specific probes labeled with ^32^P (PerkinElmer BLU008H250UC and BLU007Z001MC) and prepared by *in vitro* transcription. The non-coding RNA *SCR1* was used as a loading control. Northern blots were exposed to phosphorimager screens (Molecular Dynamics) scanned by a Typhoon 9200 (GE Healthcare, Life Sciences) and analyzed using Image Quant 5.2. All reported values are the result of at least three replicates.

*MSH4* transcripts were analyzed by RNase H treatment. Samples were prepared by combining 25 µg RNA, 3.11 µg poly(T) oligo (T_20_ by IDT) and 10 µg internal GFP cleavage probe (5′ ACA GGG CCA TCG CCA ATT GGA GTA TT) in a final volume of 25 µl. Samples were heated to 65°C for 10 min and allowed to cool to room temperature for 10 min after which 3 µl 10× reaction buffer and 2 µl RNase H (New England Biochemicals) were added. Samples were incubated at 37°C for 30 min at which point reactions were quenched by placing the samples on ice and adding an equal volume of urea loading buffer (8 M Urea, 2 mM Tris, pH 7.5, 20 mM EDTA). Samples were fractionated on a 6% denaturing polyacrylamide gel, transferred to GeneScreen Plus and analyzed by northern blotting as described above.

### RT-PCR-Seq

40 µg RNA and 6 µg poly T-oligo were combined and brought up to a volume of 31 µl with TE. The reaction was incubated at 70°C for 10 min and chilled on ice for 1–2 min. A 60 µl reaction was carried out with Superscript III (Invitrogen) with a final concentration of 1× reaction buffer 10 mM DTT, 50 µM each nucleotide and 400 U Supercript III RT. Reverse transcription was allowed to proceed for 2 h at 50°C. RNA was hydrolyzed by adding NaOH to a final concentration of 200 mM, EDTA to 50 mM and incubating at 65°C for 15 min. Reactions were neutralized by adding HEPES pH 6.5 to a final concentration of 333 µM. The fragments of interest were amplified using standard PCR methods. PCR fragments were gel-purified and re-amplified using a poly(T) oligo and a nested primer to remove artifacts. Products were sequenced at the University of Wisconsin Madison Biotechnology Center Sequencing Facility.

### Protein isolation and western blotting

Saturated overnight cultures of cells were diluted to OD_600_  =  0.1 and allowed to grow to midlog phase, OD_600_  =  0.4–0.5. During outgrowth, cells were induced with 100 µl CuSO_4_, washed with distilled water, and centrifuged. The supernatant was poured off and the pellet was flash frozen. Cell pellets were lysed by vortexing with glass beads and lysis buffer (50 mM Tris 7.4, 150 mM NaCl, 1 mM EDTA, 0.1% Tween 20, protease inhibitor cocktail (Sigma P8215), 1.742 mg/ml PMSF and 1.566 mg/ml benzamidine). Protein concentration of the clarified lysate was calculated using the Bradford method with a BSA standard. Samples were fractionated using a SDS-PAGE gel with a 12.5% resolving layer. Protein was transferred to Amersham Hybond ECL 0.45 µM (GE Healthcare). After blocking for 1 h with 1∶1 PBS:Odyssey blocking buffer (LI-COR biosciences). Membranes were probed overnight with 1∶1 PBS:Odyssey blocking buffer and 0.1% Tween-20 with both rabbit anti-GFP (Sigma-Aldrich G1544) diluted 1∶5,000 and mouse anti-FLAG M2 (Sigma F1804) diluted 1∶3,000. After washing in 1xPBS and 0.1% Tween-20 membranes were probed with both goat anti-rabbit IRDye 680LT (LI-COR 926-68021) and goat anti-mouse IRDye 800CW (LI-COR 926-32210) antibodies at 1∶25,000 dilution in 1∶1 PBS:Odyssey blocking buffer with 0.1% Tween-20 and 0.01% SDS. Membranes were then washed, allowed to dry and scanned using the Odyssey Imaging system and data were analyzed using the corresponding Image Studio software v2.1.10 (LI-COR).

## Results

### Identification of 3′-UTRs that function in NMD

**Figure 1:**
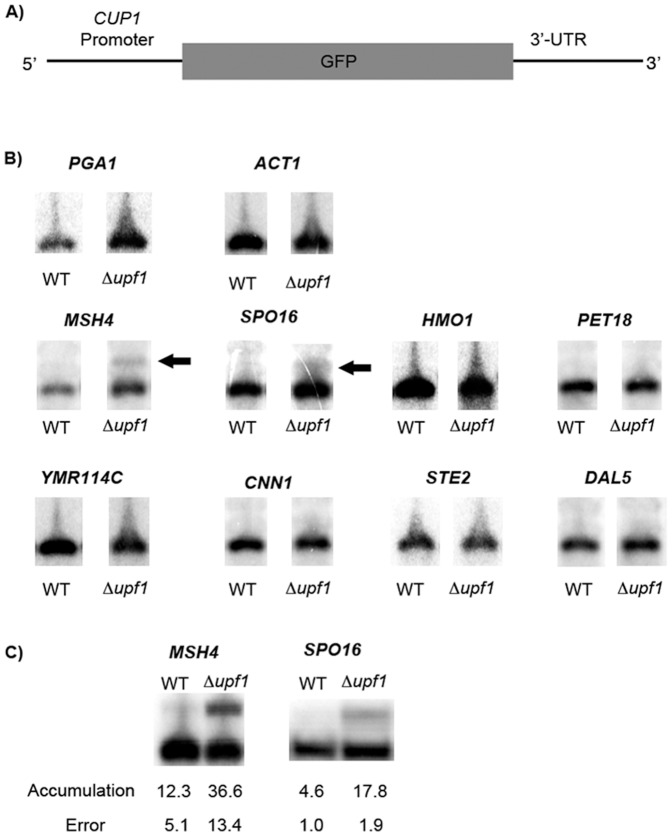
The 3′UTR of *MSH4* and *SPO16* produce transcription read-through products in an NMD dependent manner. (**A**) A reporter system was constructed on a centromeric plasmid. The reporter consisted of the 3′UTR of genes of interest fused to GFP under control of the copper inducible *CUP1* promoter. (**B**) Eight genes were examined by northern blot in a BY4741 background with both a wild type and Δupf1 strain. The 3′UTR of *PGA1* is known to target transcripts for NMD and *ACT1* is known to be insensitive to NMD. Both were included as controls [16]. Only the *MSH4* 3′UTR and *SPO16* 3′UTR displayed accumulation of a longer transcript, which are indicated by arrows. (**C**) The relative accumulation of the longer transcript was measured in a W303A background. Relative accumulation was calculated as the percentage of the longer transcript relative to total transcripts. Strain dependent differences in the magnitude of NMD-dependent accumulation in BY4741 and W303A background is consistent with previously published results [19]. All values are the result of averaging a minimum of 3 replicates.

To find 3′-UTRs that play a role in NMD, a reporter system was designed. The reporter mRNA is transcribed from a gene fusion consisting of the *CUP1* (copper metallothionine) promoter, the ORF coding for green fluorescent protein (GFP), and a 3′-UTR of choice (Fig. 1A). The reporter includes the stop codon from the 3′-UTR to be tested rather than the *GFP* stop codon so as preserve as much as possible the normal context for translation termination. The *CUP1* promoter and the *GFP* coding region are insensitive to NMD, enhancing the likelihood that NMD-dependent changes in mRNA accumulation and decay will be functionally linked to the 3′-UTR. Detection of NMD targets, normally present in low abundance, is enhanced by *CUP1*-driven expression to high levels in the presence of exogenous copper. To our knowledge, there are no reported examples where over-expression of any gene limits NMD.

After considering genomic data to select which 3′-UTRs to test (Materials and Methods), we settled on eight: *MSH4*, *SPO16, HMO1, PET18, YMR114C, CNN1, STE2*, and *DAL5*. All of the candidates were previously shown to be sensitive to NMD during vegetative growth, including *MSH4* and *SPO16*, which are known genetically to function in meiosis and sporulation [21], [22], [23].

Northern blotting was used to reveal NMD-dependent changes in reporter mRNA accumulation. Two of the eight 3′-UTRs (*MSH4* and *SPO16*) produced an altered pattern of transcripts when wild-type and *upf1Δ* strains were compared (Fig. 1B). RNA species accumulated in the Nmd^−^ strains expressing the *MSH4* and *SPO16* reporters (Fig. 1B, arrows). As shown in subsequent experiments, the longer RNAs are transcriptional read-through products that are sensitive to NMD. In the absence of NMD, the NMD-sensitive read-through transcript for *MSH4* increased from 12.3% of total transcript to 36.6%, a three-fold change. For *SPO16*, the NMD-sensitive read-through transcript increased from 4.6% to 17.8%, a four-fold change (Fig 1C).

### The *MSH4* 3′-UTR plays a role in NMD

**Figure 2:**
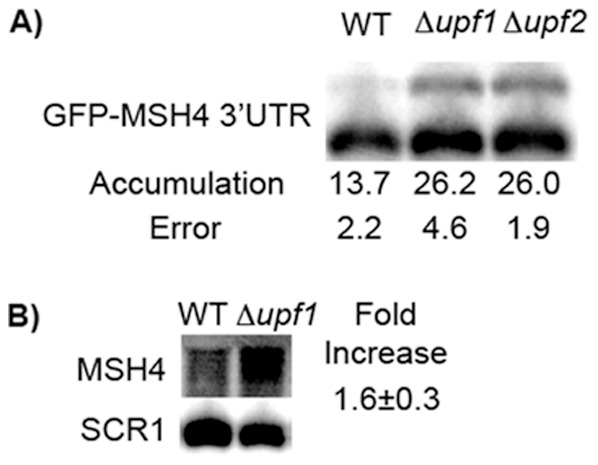
The long transcriptional read-through product from *GFP-MSH4-3*′*UTR* accumulates in an NMD-dependent manner. (**A**) The accumulation of the long *GFP-MSH4 3*′*UTR* construct was examined in a BY4741 background. The longer transcript accumulates to the same level in both a *Δupf1* and *Δupf2* strain verifying the observed effect is NMD dependent. (**B**) The endogenous *MSH4* gene shows an increase of steady state mRNA in an NMD deficient strain, confirming it as a target of NMD.

To ensure that changes in accumulation were related specifically to NMD, the experiments for the *MSH4* reporter were repeated in strains carrying *upf2*Δderived from strain BY4741. If the inactivation of *UPF1* and *UPF2* have similar effects, then the effects are most likely due to the loss of NMD. Both the *upf1Δ* and *upf2Δ* strains showed nearly identical increases in accumulation of the long *MSH4* transcript (Fig. 2A). As expected, the magnitudes of NMD-dependent differences in accumulation were lower in the BY4741 strains (Fig. 2) compared to W303A (Fig. 1) (two-fold versus three-fold, respectively) [19].

The 3′ end of the long transcript was mapped using RT-PCR-Seq (Materials and Methods). The RNA ends in a cluster of stops starting 1170-nt downstream of the *GFP* stop codon. It extends past the end of the *MSH4* 3′-UTR at 224 nucleotides into plasmid DNA, where it ends in the 3′-proximal part of the *HIS3* coding region. The *HIS3* gene is located on the plasmid downstream of *CUP1-GFP-MSH4-3*′*-UTR* reporter and is transcribed in the opposite direction. Our results define the long RNA as a transcriptional read-through product.

### The *CUP1-GFP-MSH4-3*′*-UTR* reporter produces multiple transcripts that are differentially susceptible to NMD

Previous genomic data indicated that the endogenous *MSH4* gene is expressed during vegetative growth and produces a transcript that is sensitive to NMD [13]. Before proceeding further, northern blotting was used to assess the levels of expression and to verify that *MSH4* mRNA is both expressed and sensitive to NMD in vegetative cells. A 1.6 ± 0.3 fold increase (*p* = 0.02) in endogenous *MSH4* mRNA accumulation was observed in a *upf1Δ* strain compared to a wild-type strain (Fig. 2B), indicating that *MSH4* is a likely target of NMD. This is consistent with previous NMD target studies, which define a target of NMD as an mRNA which shows a 1.5 fold or greater increase in mRNA accumulation when NMD is inhibited [13].

The expression levels of the endogenous gene were very low compared to the reporter system. In the northern blot in Fig. 2B, only one band was detected corresponding to transcripts from the endogenous gene. No equivalent to the long transcript produced from the reporter was detected, but that was expected since the long transcript from the reporter extends past the end of the 3′-UTR and into the plasmid sequences. If a long transcript of the endogenous gene is present, it accumulates at a level below detection. We opted to use the copper-inducible reporter system in further studies to over-express the transcription products and more conveniently assess the role of the 3′-UTR in targeting, but we recognize that transcripts produced from the reporter could differ in length compared to endogenous transcripts.

**Figure 3:**
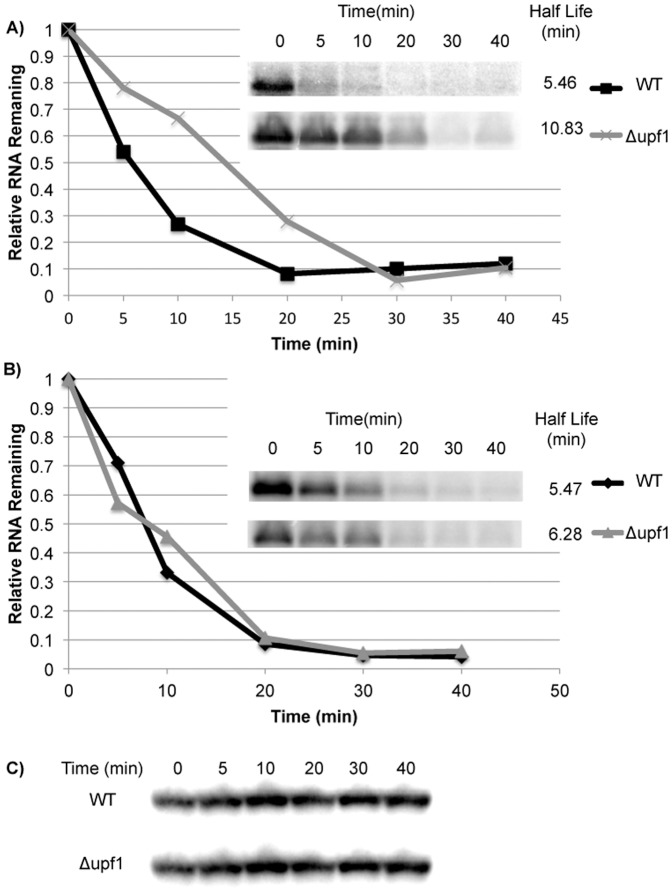
The long transcript from *GFP-MSH4-3′UTR* shows increased stability in NMD-deficient strains. Representative half-life measurements in a W303A background are shown for both (**A**) the longer transcript product and (**B**) the shorter transcript product. Inlay depicts the sample being represented in the graph and is adjacent to the corresponding marker. Only the longer transcript shows increased stability in an NMD deficient strain when transcription is inhibited by the addition of thiolutin.

Using the reporter system, we measured the half-lives of the long and short transcripts by using thiolutin to inhibit transcription followed by a time course northern blot to examine the rate of disappearance of RNA present at the time of transcription inhibition. By plotting relative RNA remaining versus time, the RNA half-life can be determined (Fig. 3). The long transcript had a two-fold increase in half-life when NMD was inactivated (5.19 ± 0.69 min in wild-type versus 11.89 ± 2.1 min in a *upf1Δ* strain) (Fig. 3A). The difference in half-life was similar to the change in accumulation at t = 0. This result confirms that the long transcript is a target of NMD. When the short transcript was examined, no differences in accumulation at t = 0 or half-life were observed (Fig. 3B). The half-lives were 5.47 ± 0.29 min in wild-type versus 6.28 ± 0.66 min in a *upf1Δ* strain), suggesting that the short transcript is not a target of NMD.

### The short transcript is a composite of five NMD-insensitive RNAs

Heterogeneity was revealed in the short transcript detected as a single band on a 1% agarose gel. The source of the heterogeneity was assessed by combining an RNase H cleavage assay with fractionation on a 6% polyacrylamide gel (Materials and Methods). By using a DNA oligonucleotide that hybridizes to the 3′ end of *GFP* and a poly(T) oligonucleotide that hybridizes to the poly(A) tail, truncated versions of the transcripts were generated by RNase H cleavage of DNA:RNA duplexes to produce RNAs that have the same 5′ end and lack a poly(A) tail. Any size differences reflect differences in length at the 3′ end.

**Figure 4:**
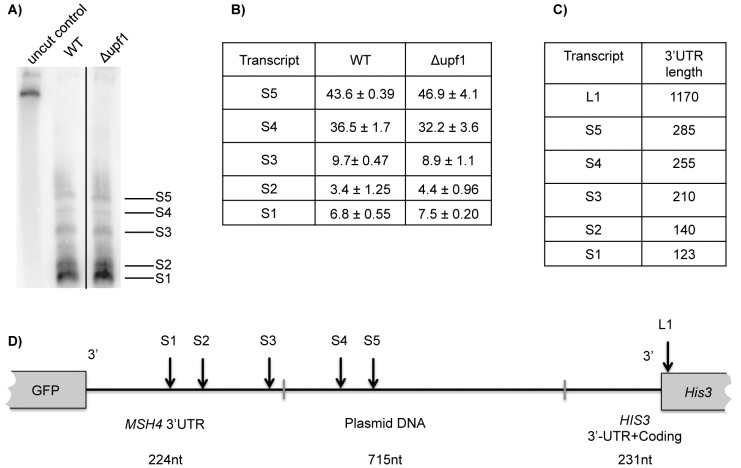
Five short transcripts are not targets of NMD. (**A**) After treatment with RNase H the shortest transcript observed on a 1% agarose gel can be resolved into 5 distinct bands on a 6% polyacrylamide gel. (**B**) Relative accumulation of the five short transcripts. Values in WT and *Δupf1* columns represent each transcript as a percentage of the sum of the first five transcripts. (**C**) Sizes of all observed transcripts from northern blots performed on polyacrylamide gels (transcripts 1-5) and agarose gels (transcript B). Sizes were determined based on a combination of RT-PCR and estimates based on relative migration in gels to known size markers. (**D**) Schematic representing the transcription products of the *GFP-MSH4 3*′*UTR* construct (not to scale). The plasmid DNA fragment is 710 bases and contains prokaryotic DNA. The *HIS3* gene is located on the crick strand relative to the *GFP* coding region.

Using this assay, five transcripts, designated S1 through S5 (Fig. 4), were detected in Nmd^+^ and Nmd^−^ strains expressing the *MSH4* reporter (Fig 4A). The resolution of bands was sufficient to separately measure changes in accumulation when NMD is inactivated. None of the five RNAs showed any NMD-dependent difference in accumulation (Fig. 4B), where accumulation was measured as a percentage of total short transcripts (all five RNAs combined). The results show that the half-life of the short transcript detected on agarose gels, which was unchanged when NMD was inactivated (Fig. 3B), is an aggregate half-life of five RNAs with different 3′ ends. Since none of the five RNAs show a NMD-dependent change in abundance, we conclude that none of the short RNA family members are targets of NMD.

The 3′ end of the shortest transcript was mapped using RT-PCR-Seq (Materials and Methods). The shortest RNA has a 3′ end 123 nucleotides from the translational stop codon in the *MSH4* reporter. The 3′ ends of the other transcripts were estimated by comparing relative migration on gels to the migration of known size markers (Fig. 4C). Transcripts S1, S2, and S3 end within the 224 nt-long *MSH4* 3′-UTR (Fig. 4D). Transcripts S4 and S5 extend into downstream plasmid DNA by 28 and 55 nucleotides respectively. As described earlier, the long NMD-sensitive transcript (L1) detected on agarose gels ends at nt 1170 (Fig. 4C). It extends 943-nt into plasmid DNA and ends in the 3′ proximal part of the *HIS3* ORF.

### NMD recruits the L1 transcript into the 5′ mRNA decay pathway

In *S. cerevisiae*, transcripts are degraded by two pathways called 5′ and 3′ decay. In 5′ decay, the 5′-cap is removed and RNA is degraded from the 5′ end by Xrn1 [24], [25]. The prevailing view is that the RNA helicase Upf1 re-models the RNA exposing the cap while simultaneously interacting with the de-capping enzyme. Consistent with this mechanism, NMD promotes de-capping and 5′ decay regardless of poly(A) tail length [25]. In 3′ decay, the RNA is degraded from the 3′ end by the exosome. Upf1 interacts with Ski7, a component of the exosome [26]. Through the interactions of Upf1 with components of both decay pathways, NMD can activate either pathway depending on the target.

**Figure 5:**
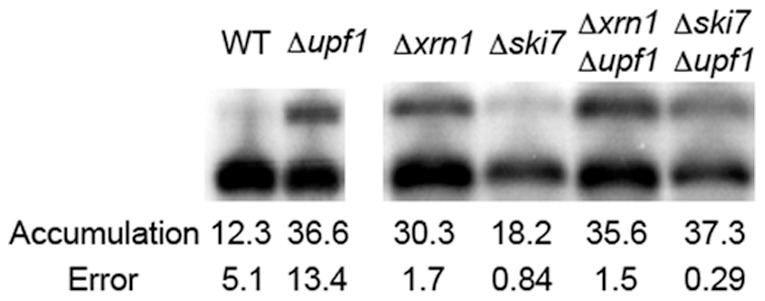
*GFP-MSH4-3*′*UTR* transcripts are degraded from the 5′ ends. The steady state mRNA accumulation levels were measured in a W303A background in *Δxrn1, Δski7, Δxrn1 Δupf1 and Δski7 Δupf1* strains. Only the *Δski7* mutant showed a statistically significant difference in accumulation from a *Δupf1* mutant. (Fig 1C) indicating that Upf1 and Xrn1 are both part of the same degradation pathway for 3′UTR-mediated NMD.

To determine which pathway predominates when the reporter transcript L1 is degraded by NMD, we used mutants to inactivate NMD and either the 5′ or the 3′ decay pathway. Null *xrn1* inactivates 5′ decay. Null *ski7* inactivates 3′ decay. Null *upf1* inactivates NMD. We analyzed the effects of blocking a decay pathway by northern blotting of RNA from strains carrying each of these single mutants and *xrn1Δ upf1Δ* and *ski7Δ upf1Δ* double mutants (Fig. 5).

The L1 transcript accumulated to a similar level in both the Δ*xrn1* and Δ*upf1* mutants (*p* = 0.16). The Δ*xrn1* Δ*upf1* and Δ*ski7* Δ*upf1* double mutants also had accumulation levels for L1 that were statistically indistinguishable from the Δ*upf1* mutant (*p* = 0.82 and *p* = 0.87, respectively). However, the Δ*ski7* mutant showed a statistically significant difference in accumulation of L1 compared to the Δ*upf1* strain (*p* = 0.001). We conclude that when NMD targets the L1 transcript for rapid decay, the 5′ decay pathway is activated predominantly over the 3′ decay pathway.

### Translation of the reporter transcripts

Premature translational stop codons play a role when NMD targets a transcript for rapid decay. When transcripts are targeted through translation of an upstream ORF or inefficient translation initiation, PTCs brought into frame trigger NMD. We tested the model that long 3′-UTRs target transcripts for NMD because the normal stop codon is recognized as a PTC. Translation termination at a PTC has been shown to be less efficient in the absence of NMD as evidenced by the accumulation of translational read-through products [27]. If the normal stop codon is seen as premature, then proteins that have C-terminal extensions should accumulate.

**Figure 6:**
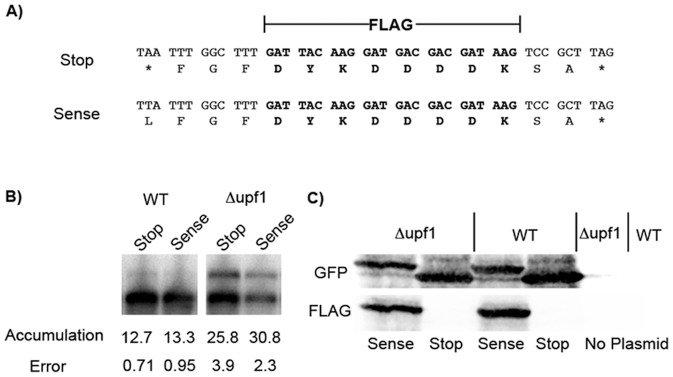
3′UTR targeting is not associated with extensive translational read-through. (**A**) A FLAG tag was inserted three codons downstream of the normal stop codon of the *GFP-MSH4 3*′*UTR* construct to monitor translational read-through. The normal TAA (stop) codon was also mutated to the sense codon TTA (Leu) as a positive control. The * indicates a stop codon. (**B**) Northern blots were performed to measure relative accumulation of the NMD sensitive transcript in a wild type and NMD deficient strain. The presence of the FLAG sequence or stop→sense mutation did not affect relative accumulation of the mRNA. (**C**) Western blot of the *GFP-FLAG-MSH4 3*′*UTR* fusion. No translational read-through product was detected in the wild type or NMD deficient strain.

To test the model, we developed an assay to detect proteins that end at the normal stop or that extend beyond the normal stop codon. A FLAG tag was inserted after the normal stop codon. As a positive control, the normal stop codon was mutated to a sense codon, Leu (Fig 6A). The FLAG tag was positioned three codons downstream of the normal stop codon and two codons upstream of the next stop codon to minimize interference with translation termination. To ensure that the FLAG sequence had no effect by itself on NMD targeting, we used northern blotting to measure accumulation of RNA produced from the mutant reporters (Fig. 6B). Neither the insertion of the FLAG sequence nor the mutation changing the stop codon to a sense codon altered the relative accumulation of the L1 transcript in wild type and Nmd^−^ strains.

Western blotting was used to assay for read-through past the normal stop codon. The expectation was that inactivation of NMD by *upf1Δ* might result in increased read-through. By using secondary antibodies that were fluorescently labeled with two different fluorophores, it was possible to analyze a single blot simultaneously for the presence of both GFP and FLAG (Fig. 1C). Four strains were assayed: two Nmd^+^ strains that have the normal stop codon or a sense codon in its place, and a similar set of two Nmd^−^ strains. GFP was expressed in all of the strains. The immediate downstream stop codon after the FLAG tag is an efficient terminator since only one band was detected when examining FLAG or GFP. FLAG-tagged protein was detected when the normal stop codon was mutated to a sense codon. In Nmd- strains, no FLAG-tagged protein that extends past the normal site for translation termination was detected.

To increase the sensitivity of the experiment, we switched from a single-copy centromeric plasmid to a multi-copy 2 µ plasmid that over-expresses the reporter RNAs on a per cell basis. Using the centromeric plasmid system, we can detect 8–10% read-through. Using the 2 µ plasmid, sensitivity improves to about 1% read-through. These read-through values for theoretical limits of detection were calculated by comparing the signal for sense codon construct, defined as 100% read-through, with background, defined as 0% read-through. Furthermore, the 1% threshold for 2 µ plasmid expression is based on translation of all reporter transcripts. Since Nmd-sensitive L1 comprises about one third of total reporter transcripts in Nmd^−^ strains, read-through would have to occur at about 3% or more in order to be detected. No read-through proteins were detected (data not shown).

In all of these experiments the accumulation levels observed for tagged proteins suggest that any read-through products that might arise should have the same stability as the normally terminated protein. It is therefore unlikely that read-through proteins are made and then degraded. Although the normal stop codon is considered the best candidate to trigger NMD, the associated translational read-through typical of PTCs in Nmd^−^ strains either does not occur with the normal stop codon or read-through is below the 3% threshold.

## Discussion

To identify transcripts targeted for NMD through the 3′-UTR, we fused the 3′-UTRs from candidate genes with green fluorescent protein (GFP) expressed from a copper-inducible *CUP1* promoter on a centromeric plasmid. Northern blotting was used to monitor transcript length and abundance in Nmd^+^ and Nmd^−^ strains. The most likely candidates for 3′-UTR-mediated targeting were gleaned from genomic data identifying direct targets of NMD (Guan et al., 2006). After subtracting those known or suspected of being targeted without 3′-UTR involvement and by considering the predicted length of the 3′-UTRs, we settled on a short list of eight candidate genes. Two of the 3′-UTRs targeted a reporter transcript for NMD (*MSH4* and *SPO16*). Both reporters produced long NMD-sensitive read-through transcripts. The remaining six 3′-UTRs failed to target the reporter transcripts for NMD possibly due to false discoveries and misclassifications of direct and indirect targets of NMD inherent in the genomic data, erroneous predictions of the targeting mechanism for a given transcript, or erroneous predictions of 3′-UTR length leading to reporters with incomplete 3′-UTRs. The detection of both NMD-sensitive and NMD-insensitive *MSH4* 3′-UTR transcripts provides unique internal controls permitting examination of stop codon context, 3′-UTR sequence requirements, and 3′-UTR length requirements for NMD targeting.

Since yeast lack a defined polyadenylation regulatory sequence and the reporter construct produces transcripts that extend well past the MSH4 3′-UTR sequence it is possible that we could be observing an effect related to transcription termination and post-transcriptional processing. We find this possibility highly unlikely since yeast mutants with defects in transcription termination and processing also show an inability to export the improperly processed RNA from the nucleus [28]. NMD occurs in the cytoplasm during translation so any defect in transcription termination or processing would be unable to target a defective transcript for NMD.

In depth studies of the *GFP-MSH4 3*′*-UTR* reporter showed that only the longest transcript is a target of NMD while five shorter transcripts are insensitive to NMD. To our knowledge this is the first known example of a gene producing multiple transcripts in which only a subset are targets of NMD in *S. cerevisiae*. All other targeting mechanisms involve recognition of a PTC by components of the surveillance complex, including Upf proteins and translation termination factors that mediate the steps in NMD leading to rapid RNA decay. For targeting by a 3′- UTR, the most logical stop codon recognized by the surveillance complex is the normal stop codon by virtue of its proximity to the 3′-UTR. If the context surrounding the normal stop codon had an effect on 3′-UTR-mediated targeting, we should have observed changes in accumulation and half-life for all of the transcripts produced from the *GFP-MSH4-3*′*UTR* reporter in Nmd^−^ strains. Instead, we found that the long L1 transcript was sensitive to NMD based increased accumulation and decreased decay rate in Nmd^−^ strains, but the five shorter transcripts, S1 through S5, were insensitive to NMD. In the aggregate, there was no change in abundance or half-life of the short transcripts in Nmd^−^ strains. Since the short and long transcripts share a stop codon with identical context we can confidently state that the context of the stop codon has no effect on NMD targeting via the 3′-UTR.

3′ end mapping showed that the S1, S2, and S3 transcripts terminated within the *MSH4-3*′*-UTR*, whereas S4, S5, and L1 extended beyond the 3′-UTR into adjacent plasmid sequences. Since L1 is NMD-sensitive but S4 and S5 are insensitive, plasmid sequences in the transcripts up to the S5 3′ end are not *per se* responsible for NMD-targeting. L1 terminates near the 3′ end of the *HIS3* ORF on the plasmid. Since *HIS3* is present in opposite orientation to the reporter gene, it is possible but unlikely that the portions of the *HIS3* gene included in the L1 transcript contain signals for NMD targeting. The most likely model is that targeting is dependent on the length of the 3′-UTR. Since poly(A) binding protein plays a role in translation termination at the normal stop codon [29], long 3′-UTRs might disrupt the termination process due to the extended distance between the stop codon and poly(A) binding protein bound to the poly(A) tail. Put another way, the normal stop codon might be recognized as a premature stop codon by the surveillance complex when the 3′-UTR exceeds a maximum length required for efficient translation termination.

Endogenous *MSH4* is a target of NMD (Guan et al., 2006), but the endogenous gene cannot express a transcript equivalent to the NMD-sensitive L1 transcript from the reporter, which includes plasmid sequences. However, RNA-seq experiments show that endogenous *MSH4* produces multiple transcripts with different 3′ ends [30], [31]. We confirmed that *MSH4* is a target of NMD by northern blotting, but could not resolve potential multiple transcripts on agarose gels even when the endogenous MSH4 is placed under the inducible *CUP1* promoter and overexpressed (data not shown). We estimate transcripts that differ in length by up to 500 nt would migrate as a single, unresolved band. Resolution by PAGE was deemed unreliable due to the extremely low abundance of *MSH4* transcripts in vegetative cells. We have therefore not been able to confirm that endogenous *MSH4* is targeted for NMD by a long 3′-UTR.

Although we did not perform a detailed analysis of the role of the *SPO16 3*′*-UTR* in NMD, we believe that a phenomenon is occurring similar to that observed for *MSH4*. Detailed studies have shown that *SPO16* produces four transcripts with 3′-UTRs ranging from 147 to 747 nt in length [32]. From our studies of the *MSH4 3*′*-UTR*, transcript S5, which is 285 nt in length, did not trigger NMD. If length is the only important parameter for NMD targeting, some of the *SPO16* transcripts are too short to trigger NMD while others could be long enough and, according to our data, at least one is NMD-sensitive. We have no reason to believe that the *MSH4-* and *SPO16* 3′-UTRs target reporter transcripts for NMD because the endogenous genes perform functions in sporulation. In vegetative cells, both genes are expressed albeit at low levels, and might perform vegetative functions. Studies based on our reporter system cannot address whether NMD impacts gene expression in vegetative cells, sporulating cells, or both.

Transcripts with long 3′-UTRs from any gene could be generated by inefficient termination of transcription or aberrant processing by the cleavage/polyadenylation complex, suggesting the possibility that transcripts with long 3′-UTRs are targets of NMD because NMD is performing an RNA surveillance function ultimately related to the efficacy of transcription termination. The link that would make this model attractive is if long 3′-UTRs caused a disruption of translation termination at the normal stop codon. While other targeting mechanisms prevent the accumulation of truncated proteins, disruption of translation termination at the normal stop codon by a long 3′-UTR might lead to translational read-through and the accumulation of proteins that are longer than normal. NMD might degrade abnormally long and potentially deleterious transcripts as an RNA surveillance mechanism.

To test this model, we designed a western blotting assay based on the inclusion of epitope tags in *MSH4 3*′*-UTR* reporters so that proteins that end at the normal stop codon could be detected and distinguished from abnormally long read-through proteins. According to our calculations, read-through would have to occur at a threshold of about 3% in NMD sensitive transcripts when expressed from a multi-copy 2 µ plasmid in order for the read-through proteins to be detected. The mixture of target and non-target transcripts prevented us from achieving higher sensitivity. No translational read-through was detected.

For premature stop codons embedded in a dual reporter, the frequency of translational read-through past a PTC can be as high as 2–3% in Nmd^−^ strains [27]. Read-through levels were shown to depend on which stop codon is used, where it is located, and the context surrounding the stop codon. Since no *MSH4 3*′*-UTR*-mediated translational read-through was detected using our immunological assay, we conclude either that the proposed link between errors in transcription termination and translation termination is invalid or that the error rates for translation termination are too low to detect using this system.

We investigated whether temperature-sensitive mutants that disrupt transcription termination or processing could be used to study the potential link between transcription termination and translation termination as related to NMD. However, all known mutants that disrupt transcription termination or processing also disrupt RNA export, which precludes the ability to determine whether mutant-induced transcriptional read-through transcripts are sensitive to NMD. Further studies will be required to resolve the question of whether 3′-UTRs are part of an RNA surveillance system to monitor transcription termination or whether programmed inefficiencies in transcription termination bring a subset of endogenous transcripts under the control of NMD.

Both 5′- and 3′-UTRs have been implicated in targeting mechanisms [14], [15], [16]. Heterogeneity of 5′ and 3′ ends are expected to impact NMD allowing some transcripts from a given gene to be prone to NMD while others are insensitive. For example, multiple start sites for transcription initiation can produce transcripts with longer 5′-UTRs that contain a uORF while other transcripts with shorter 5′-UTRs might lack the start codon for a uORF. 25% of yeast transcripts have heterogeneous 5′ ends [33]. Transcripts in multicellular eukaryotes exhibit similar heterogeneity. For example, 10–18% of mammalian genes have heterogenous 5′ ends [34].

Similarly, we propose that inefficient transcription termination could produce transcripts that have short and long 3′-UTRs that are differentially prone to NMD. Close to half of yeast genes produce multiple transcripts that vary in 3′-UTR length and ∼700 genes produce transcript isoforms that differ in poly(A) tail position by at least 100 bases [35]. We therefore surmise that the phenomenon observed in the GFP-*MSH4* 3′-UTR reporter system is not likely to be a stand-alone example of differential targeting, but rather, might be representative of a larger subset of genes that produce multiple transcripts that are differentially prone to NMD. If this is the case, transcripts that produce long 3′-UTRs due to inefficient transcription termination might be recruited into the NMD pathway for regulatory purposes. Also, NMD might perform an RNA surveillance function to eliminate transcripts that extend beyond the normal transcription termination site due to errors in transcription termination.

## References

[1] Culbertson MR, Leeds PF (2003) Looking at mRNA decay pathways through the window of molecular evolution. Curr Opin Genet Dev 13: 207–214. 12672499 10.1016/s0959-437x(03)00014-5

[2] Leeds P, Peltz SW, Jacobson A, Culbertson MR (1991) The product of the yeast UPF1 gene is required for rapid turnover of mRNAs containing a premature translational termination codon. Genes Dev 5: 2303–2314. 1748286 10.1101/gad.5.12a.2303

[3] Leeds P, Wood JM, Lee BS, Culbertson MR (1992) Gene products that promote mRNA turnover in Saccharomyces cerevisiae. Mol Cell Biol 12: 2165–2177. 1569946 10.1128/mcb.12.5.2165-2177.1992 PMC364388

[4] Chamieh H, Ballut L, Bonneau F, Le Hir H (2008) NMD factors UPF2 and UPF3 bridge UPF1 to the exon junction complex and stimulate its RNA helicase activity. Nat Struct Mol Biol 15: 85–93. 18066079 10.1038/nsmb1330

[5] Czaplinski K, Ruiz-Echevarria MJ, Paushkin SV, Han X, Weng Y, et al (1998) The surveillance complex interacts with the translation release factors to enhance termination and degrade aberrant mRNAs. Genes Dev 12: 1665–1677. 9620853 10.1101/gad.12.11.1665 PMC316864

[6] He F, Brown AH, Jacobson A (1997) Upf1p, Nmd2p, and Upf3p are interacting components of the yeast nonsense-mediated mRNA decay pathway. Mol Cell Biol 17: 1580–1594. 9032286 10.1128/mcb.17.3.1580 PMC231884

[7] Atkin AL, Altamura N, Leeds P, Culbertson MR (1995) The majority of yeast UPF1 co-localizes with polyribosomes in the cytoplasm. Mol Biol Cell 6: 611–625. 7545033 10.1091/mbc.6.5.611 PMC301219

[8] Wang W, Czaplinski K, Rao Y, Peltz SW (2001) The role of Upf proteins in modulating the translation read-through of nonsense-containing transcripts. Embo J 20: 880–890. 11179232 10.1093/emboj/20.4.880 PMC145432

[9] Atkin AL, Schenkman LR, Eastham M, Dahlseid JN, Lelivelt MJ, et al (1997) Relationship between yeast polyribosomes and Upf proteins required for nonsense mRNA decay. J Biol Chem 272: 22163–22172. 9268361 10.1074/jbc.272.35.22163

[10] Swisher KD, Parker R (2011) Interactions between Upf1 and the decapping factors Edc3 and Pat1 in Saccharomyces cerevisiae. PLoS One 6: e26547. 22065998 10.1371/journal.pone.0026547 PMC3204985

[11] Min EE, Roy B, Amrani N, He F, Jacobson A (2013) Yeast Upf1 CH domain interacts with Rps26 of the 40S ribosomal subunit. Rna 19: 1105–1115. 23801788 10.1261/rna.039396.113 PMC3708530

[12] Mitchell P, Tollervey D (2003) An NMD pathway in yeast involving accelerated deadenylation and exosome-mediated 3′-->5′ degradation. Mol Cell 11: 1405–1413. 12769863 10.1016/s1097-2765(03)00190-4

[13] Guan Q, Zheng W, Tang S, Liu X, Zinkel RA, et al (2006) Impact of nonsense-mediated mRNA decay on the global expression profile of budding yeast. PLoS Genet 2: e203. 17166056 10.1371/journal.pgen.0020203 PMC1657058

[14] Gaba A, Jacobson A, Sachs MS (2005) Ribosome occupancy of the yeast CPA1 upstream open reading frame termination codon modulates nonsense-mediated mRNA decay. Mol Cell 20: 449–460. 16285926 10.1016/j.molcel.2005.09.019

[15] Welch EM, Jacobson A (1999) An internal open reading frame triggers nonsense-mediated decay of the yeast SPT10 mRNA. Embo J 18: 6134–6145. 10545123 10.1093/emboj/18.21.6134 PMC1171677

[16] Kebaara BW, Atkin AL (2009) Long 3′-UTRs target wild-type mRNAs for nonsense-mediated mRNA decay in Saccharomyces cerevisiae. Nucleic Acids Res 37: 2771–2778. 19270062 10.1093/nar/gkp146 PMC2685090

[17] Muhlrad D, Parker R (1999) Aberrant mRNAs with extended 3′ UTRs are substrates for rapid degradation by mRNA surveillance. Rna 5: 1299–1307. 10573121 10.1017/s1355838299990829 PMC1369852

[18] Sureau A, Gattoni R, Dooghe Y, Stevenin J, Soret J (2001) SC35 autoregulates its expression by promoting splicing events that destabilize its mRNAs. Embo J 20: 1785–1796. 11285241 10.1093/emboj/20.7.1785 PMC145484

[19] Kebaara B, Nazarenus T, Taylor R, Atkin AL (2003) Genetic background affects relative nonsense mRNA accumulation in wild-type and upf mutant yeast strains. Curr Genet 43: 171–177. 12695845 10.1007/s00294-003-0386-3

[20] Hurowitz EH, Brown PO (2003) Genome-wide analysis of mRNA lengths in Saccharomyces cerevisiae. Genome Biol 5: R2. 14709174 10.1186/gb-2003-5-1-r2 PMC395734

[21] Novak JE, Ross-Macdonald PB, Roeder GS (2001) The budding yeast Msh4 protein functions in chromosome synapsis and the regulation of crossover distribution. Genetics 158: 1013–1025. 11454751 10.1093/genetics/158.3.1013 PMC1461720

[22] Shinohara M, Oh SD, Hunter N, Shinohara A (2008) Crossover assurance and crossover interference are distinctly regulated by the ZMM proteins during yeast meiosis. Nat Genet 40: 299–309. 18297071 10.1038/ng.83

[23] Nishant KT, Chen C, Shinohara M, Shinohara A, Alani E (2010) Genetic analysis of baker's yeast Msh4-Msh5 reveals a threshold crossover level for meiotic viability. PLoS Genet 6: e1001083. 20865162 10.1371/journal.pgen.1001083 PMC2928781

[24] Beelman CA, Stevens A, Caponigro G, LaGrandeur TE, Hatfield L, et al (1996) An essential component of the decapping enzyme required for normal rates of mRNA turnover. Nature 382: 642–646. 8757137 10.1038/382642a0

[25] Muhlrad D, Parker R (1994) Premature translational termination triggers mRNA decapping. Nature 370: 578–581. 8052314 10.1038/370578a0

[26] Takahashi S, Araki Y, Sakuno T, Katada T (2003) Interaction between Ski7p and Upf1p is required for nonsense-mediated 3′-to-5′ mRNA decay in yeast. Embo J 22: 3951–3959. 12881429 10.1093/emboj/cdg374 PMC169047

[27] Keeling KM, Lanier J, Du M, Salas-Marco J, Gao L, et al (2004) Leaky termination at premature stop codons antagonizes nonsense-mediated mRNA decay in S. cerevisiae. Rna 10: 691–703. 15037778 10.1261/rna.5147804 PMC1262634

[28] Brodsky AS, Silver PA (2000) Pre-mRNA processing factors are required for nuclear export. Rna 6: 1737–1749. 11142374 10.1017/s1355838200001059 PMC1370044

[29] Cosson B, Couturier A, Chabelskaya S, Kiktev D, Inge-Vechtomov S, et al (2002) Poly(A)-binding protein acts in translation termination via eukaryotic release factor 3 interaction and does not influence [PSI(+)] propagation. Mol Cell Biol 22: 3301–3315. 11971964 10.1128/MCB.22.10.3301-3315.2002 PMC133780

[30] Johnson SA, Kim H, Erickson B, Bentley DL (2011) The export factor Yra1 modulates mRNA 3′ end processing. Nat Struct Mol Biol 18: 1164–1171. 21947206 10.1038/nsmb.2126 PMC3307051

[31] Pelechano V, Wei W, Steinmetz LM (2013) Extensive transcriptional heterogeneity revealed by isoform profiling. Nature 497: 127–131. 23615609 10.1038/nature12121 PMC3705217

[32] Malavasic MJ, Elder RT (1990) Complementary transcripts from two genes necessary for normal meiosis in the yeast Saccharomyces cerevisiae. Mol Cell Biol 10: 2809–2819. 2188099 10.1128/mcb.10.6.2809-2819.1990 PMC360642

[33] Rojas-Duran MF, Gilbert WV (2012) Alternative transcription start site selection leads to large differences in translation activity in yeast. Rna 18: 2299–2305. 23105001 10.1261/rna.035865.112 PMC3504680

[34] Araujo PR, Yoon K, Ko D, Smith AD, Qiao M, et al (2012) Before It Gets Started: Regulating Translation at the 5′ UTR. Comp Funct Genomics 2012: 475731. 22693426 10.1155/2012/475731 PMC3368165

[35] Wilkening S, Pelechano V, Jarvelin AI, Tekkedil MM, Anders S, et al (2013) An efficient method for genome-wide polyadenylation site mapping and RNA quantification. Nucleic Acids Res 41: e65. 23295673 10.1093/nar/gks1249 PMC3597643

